# Correction to: DeepFormer: a hybrid network based on convolutional neural network and flow-attention mechanism for identifying the function of DNA sequences

**DOI:** 10.1093/bib/bbae181

**Published:** 2024-04-15

This is a correction to: Zhou Yao, Wenjing Zhang, Peng Song, Yuxue Hu, Jianxiao Liu, DeepFormer: a hybrid network based on convolutional neural network and flow-attention mechanism for identifying the function of DNA sequences, *Briefings in Bioinformatics*, Volume 24, Issue 2, March 2023, bbad095, https://doi.org/10.1093/bib/bbad095.

In the originally published version of this manuscript the first affiliation for author Zhou Yao was inadvertently omitted from the online version of the paper. This has now been corrected online.

